# The prevalence of hazardous and harmful drinking in the UK Police Service, and their co-occurrence with job strain and mental health problems

**DOI:** 10.1017/S2045796021000366

**Published:** 2021-06-21

**Authors:** P. Irizar, S. H. Gage, M. Field, V. Fallon, L. Goodwin

**Affiliations:** 1 Department of Psychology, Institute of Population Health, University of Liverpool, Liverpool, UK; 2 Department of Psychology, University of Sheffield, Sheffield, UK

**Keywords:** Epidemiology, hazardous alcohol use, job strain, mental health

## Abstract

**Aims:**

Due to the stressful nature of policing, police employees are at risk of mental health problems and problematic alcohol use. We aim to determine the prevalence of hazardous and harmful alcohol use in the UK Police Service, and to explore the associations with job strain and mental health problems.

**Methods:**

Cross-sectional data from the Airwave Health Monitoring Study (*N* = 40 986) included measures of alcohol consumption (total units in past week), mental health (depression, anxiety and post-traumatic stress disorder [PTSD]) and job strain. The associations between mental health and job strain with alcohol consumption (i.e. abstinence, low-risk [<14 units per week, reference group], hazardous [>14 to 35 units for women, >14 to 50 units for men], harmful [>35 units for women, >50 units for men]), were analysed using multinomial logistic regressions, adjusting for potential confounders (i.e. age, gender, ethnicity, marital status, children under 18, income and smoking status).

**Results:**

A total of 32.6% of police employees reported hazardous drinking, with 3.0% drinking at harmful levels. Compared to those without a mental health problem, police employees with depression, anxiety or PTSD were twice as likely to be harmful drinkers and were also 1.3 times more likely to report abstinence. Those reporting low strain (reference group) were more likely to drink hazardously compared to those reporting high strain, which was statistically moderated by mental health. When the sample was stratified by mental health status, the association between low strain (compared to all other categories) and hazardous drinking, was significant only in those without a mental health problem.

**Conclusions:**

These findings indicate that police employees may be an occupational group at risk of alcohol harm, with one-third drinking hazardously. The J-shaped relationship between mental health and alcohol use highlights a need for an integration of mental health and alcohol services, tailored for the UK Police Service.

## Introduction

Policing is a stressful occupation, characterised by operational stressors such as frequent exposure to traumatic incidents (Tuckey *et al*., 2012), and organisational stressors such as cuts to budgets, reducing officer numbers and increasing demands (Elliott-Davies, 2019); Allen and Audickas, 2020). UK Police Federation surveys identified that 67% of police employees reported an inability to meet demands and 60% reported low morale (Boag-Munroe, 2016; Elliott-Davies, 2019)). However, the surveys' low response rates could indicate a sampling bias towards those experiencing problems, which is common in occupational studies (Goodwin *et al*., 2013). Nevertheless, exposure to operational and organisational stressors increases the risk of mental health problems (van der Velden *et al*., 2010; Houdmont and Randall, 2016), which could lead to maladaptive coping behaviours, such as hazardous drinking (a pattern of alcohol consumption that increases someone's risk of harm) or harmful drinking (a pattern of alcohol consumption that is causing mental or physical damage) (Lindsay and Shelley, 2009; Brough *et al*., 2016).

Data from 4193 Australian police officers, two decades ago, found that 32% met the criteria for hazardous drinking and 3% for harmful drinking (Davey *et al*., 2000*a*, 2000*b*), compared to just 12.5% of the Australian general population drinking hazardously or harmfully (National Drug Strategy, 1996). Certain sociodemographic factors were associated with hazardous and harmful drinking: male gender, younger age, being single, lower education, smoking and holding a lower job role (Davey *et al*, 2000a, Davey *et al*., 2001, Obst and Davey, 2003). More recent evidence from the United States (USA), estimated the prevalence of hazardous drinking to be 16% (similar to the US general population), although the sample size was relatively small (Ballenger *et al*., 2011). Additional literature identified that traumatic stressors increased the likelihood of hazardous drinking in officers (Violanti *et al*., 2011; Chopko *et al*., 2013).

Alcohol and mental health problems often co-occur, with several population-based studies demonstrating this relationship (Regier *et al*., 1990; Kessler *et al*., 1997; Burns and Teesson, 2002; Jane-Llopis *et al*., 2006). Within the UK general population, individuals with a mental health problem are twice as likely to have an alcohol problem, compared to those with no mental health problem (Farrell *et al*., 2001; Davis *et al*., 2020; Puddephatt *et al*., 2020). Contrarily, those with mental health problems are also more likely to abstain from alcohol (Goodwin *et al*., 2017), as they may avoid alcohol to prevent further mental health decline (Strid *et al*., 2018). It is well established that UK military personnel have higher levels of alcohol problems compared to the general population (Fear *et al*., 2007), with high comorbidity with post-traumatic stress disorder (PTSD) (Head *et al*., 2016) and common mental disorders (Goodwin *et al*., 2017). Comparable levels of alcohol problems and comorbid mental health problems are predicted in UK police employees, due to occupational similarities, e.g. high-trauma exposure and a male-dominated culture which promotes risk-taking behaviours (Hales *et al*., 2015).

Job strain is a further risk factor for heavy drinking (Crum *et al*., 1995; Violanti *et al*., 2011), which may be exacerbated in UK police employees, given recent cuts (Boag-Munroe, 2016; Allen and Zayed, 2019). According to the strain hypothesis of job demand-control (JDC), those working in a high strain job (high demands and low control, i.e. an inability to set own goals and priorities) experience the lowest well-being (Karasek, 1979). This model has been expanded to the JDC-Support model, translated to the iso-strain hypothesis, whereby workers in an iso-strain job (i.e. high demands, low control and low support) have the most negative outcomes (Van der Doef and Maes, 1999; Häusser *et al*., 2010) as high support moderates the negative impact of high strain on well-being. In relation to alcohol use in police officers, early literature demonstrates a relationship between high strain and heavy drinking (Violanti *et al*., 1985; Kohan and O'connor, 2002; Violanti *et al*., 2011).

The current study aims to (i) determine the prevalence of hazardous and harmful alcohol use and frequent binge drinking (six or more drinks on one occasion, at least twice a month) in UK police employees; (ii) explore the associations between probable mental health (i.e. depression, anxiety and PTSD) and job strain, with alcohol use and (iii) examine whether the associations between job strain and alcohol use differ by the level of support or mental health status. It is hypothesised that those with a probable mental health problem or high job strain will be more likely to drink hazardously/harmfully or abstain from alcohol. It is expected that the association between high job strain and hazardous/harmful drinking will be stronger in individuals with lower support or a probable mental health problem.

This study is pre-registered on Open Science Framework, where the *a priori* research questions and hypotheses are outlined in more detail: DOI 10.17605/OSF.IO/T8EKJ.

## Methods

### Study sample

The study sample is the Airwave Health Monitoring Study (Elliott *et al*., 2014), which was open to all police forces across the UK. Baseline data were collected between June 2006 and March 2015 and this analysis is based on a sample of 40 986 police employees. A pilot study was completed in 2006 in one force (representing 6% of the total sample) and this version of the protocol did not include a measure of PTSD. Recruitment was then rolled out across the 28 participating forces (out of 54), recruiting approximately 3000–6000 participants each year (except for 2015, where only 1674 participants were recruited). The response rate averaged 50% across participating forces (range: 6–74%). At the time of recruitment, there were a total of 259 283 police employees in the UK; the present sample represents approximately 16% of the target population (Hargreaves *et al*., 2016). The ethnicity of the sample is representative, as 95% of police employees were White in the overall Police Service and in the sample, and the gender composition of the sample is also representative (Allen and Audickas, 2020).

### Data collection

The Airwave Health Monitoring Study was established to determine possible health risks associated with the use of Terrestrial Trunked Radio (TETRA), a digital communication system used by police forces since 2001 (Elliott *et al*., 2014), and wider health issues. Data were collected via an enrolment questionnaire through administration or occupational health services and health screens conducted by trained nurses. The study measured sociodemographics, occupational variables, TETRA usage, medical history, physical health, blood and urine samples, diet and alcohol use and some mental health variables. The Airwave Health Monitoring Study design and protocol have been described in detail elsewhere (Elliott *et al*., 2014).

### Measures

The outcome variable was categories of alcohol use, used by the Health Survey for England (NHS Digital, 2018), based on the UK Chief Medical Officer's guidelines for low-risk drinking (0–14 units) (Department of Health and Social Care, 2016) and National Institute for Health and Care Excellence (NICE) guidance for hazardous use (above 14 to 35/50 units for women/men), and harmful use (above 35/50 units for women/men) (NICE, 2014). Participants were asked if they drink alcohol, those who answered ‘no’, were defined as ‘non-drinkers’. Remaining participants completed a past weekly drinks diary, for the following: red wine, white wine, fortified wine, spirits, beer (converted into units). One item measured binge drinking (i.e. six or more drinks on one occasion). Participants who reported binge drinking at least two to four times a month were coded as ‘frequently binge drinks’.

The explanatory variables were measures of mental health and job strain. Symptoms of depression were measured using the 9-item Patient Health Questionnaire (PHQ-9), with a validated cut-off of 10 indicating probable depression (range from 0 to 27) (Kroenke *et al*., 2001). Responses were given on a 4-point Likert scale ranging from ‘not at all’ to ‘nearly every day’. Symptoms of anxiety were measured using the 7-item anxiety subscale of the Hospital Depression and Anxiety Score (HADS-A), with a validated cut-off of 11 indicating probable anxiety (scores range from 0 to 21) (Zigmond and Snaith, 1983). Probable PTSD was measured using the 10-item Trauma Screen Questionnaire (TSQ). Responses are usually binary (yes or no), but a 4-point Likert scale was used (‘not at all’ to ‘extremely’), therefore ‘not at all’ was coded as 0 and remaining responses were coded as 1, using a cut-off of 6 (scores range from 0 to 10) (Brewin *et al*., 2002). The TSQ was only administered if participants reported a traumatic experience in the past 6 months (*N* = 5539), coding the remaining participants as ‘non-case’ for PTSD.

Job strain was measured using ten items of the Job Content Questionnaire (JCQ) (Karasek, 1985), with four items measuring support (e.g. when having difficulties at work, I get support from colleagues), four items measuring control (e.g. I have a lot of say about what I do) and two items measuring demand (e.g. I have an excessive amount of work to do). A quadrant approach (combining demand with control) was used and participants were grouped into high (high demand, low control), low (low demand, high control), active (high demand, high control) and passive (low demand, low control) strain, using the sample median scores (Gibson *et al*., 2018). Support was kept continuous (ranging from 4 to 16) as the data were skewed towards higher support (i.e. median = 14).

Sociodemographic measures included gender, age, country (England, Wales and Scotland), marital status, ethnicity, education and number of children under 18. Occupational measures included years in the police force, police role (police officer, police staff, other – police officers are those who have completed a 2-year probationary period and become a serving officer; police staff refers to a range of roles, such as intelligence analysts, administration, response call operators, custody and detention staff) and income. Health measures included days of sickness absence in the past year and smoking status.

### Data analysis

Descriptive statistics (frequencies and percentages, with 95% confidence intervals [CIs]) were reported for sample characteristics and each category of alcohol consumption, binge drinking, probable mental health problems and job strain. Median units with interquartile range (IQR) were reported for categories of alcohol consumption and binge drinking.

Unadjusted and adjusted logistic or multinomial logistic regressions determined the associations between probable depression, anxiety and PTSD, with alcohol use (reference group: low risk) and binge drinking as outcomes. The regressions were adjusted for an *a priori* predefined list of potential confounders believed to be associated with alcohol use: age, gender, ethnicity, marital status, children under 18, income and smoking status.

Unadjusted and adjusted logistic or multinomial logistic regressions examined the associations between job strain and alcohol use or binge drinking, adjusting for the same potential confounders. Two interaction terms were created (job strain × job support, job strain × mental health), to determine whether job support and/or the presence of any mental health problem moderated the relationship between job strain and alcohol use.

Exploratory logistic regressions or multinomial logistic regressions, which adjusted for age and gender, examined the sociodemographic, occupational and health associations with alcohol use and binge drinking as the outcomes. Exploratory sensitivity analysis of non-drinkers examined mental health differences in never drinkers *v*. former drinkers, using unadjusted and adjusted logistic regression analyses. Exploratory descriptive statistics, presenting key explanatory variables and the outcome variable, separated by year of data collection, were conducted to explore cross-sectional trends over time, and are reported and discussed in the online Supplementary materials.

All statistical analyses were conducted in the statistical package STATA SE 15 (Stata Corporation, 2017).

### Missing data

The proportion of missing data for the variables of interest was less than 1%, except for PTSD (6%, *N* = 2469) and police role (9%, *N* = 3859). Police role was only included in the online Supplementary analysis, exploring the demographic associations with alcohol use (online Supplementary Table S1). PTSD was only included as an explanatory variable when looking at the association between mental health and alcohol use, with a final sample size of 38 517 for this analysis.

### Ethics

The Airwave Health Monitoring Study received ethical approval from the National Health Service multi-site research ethics committee (MREC/13/NW/0588). Written informed consent was obtained from all participants.

## Results

### Sample characteristics

The sample characteristics (*N* =  40 986) are described in Table 1. A total of 70.1% of the sample were police officers and 27.9% were police staff. Only 20.7% of the sample had served for less than 5 years, at the time of enrolment into the study, and 45.8% had been serving for more than 20 years. Few police employees reported an income above £60 000 (3.2%), with the majority earning between £26 000 and £37 999. Men made up 62.9% of the sample. Approximately 38.8% of the sample were aged 40–49, with the mean age being 40.55 (±8.92). Almost 95% of the sample were White and 76.5% of the sample obtained qualifications higher than GSCEs or equivalent. Almost 10% were current smokers.

**Table 1.** Sociodemographic, occupational and health characteristics of participants (*N* =  40 986)

Characteristic	Complete *N* (% missing)	*N*	%	95% CI
Gender	40 986 (0.00)			
Men		25 788	62.92	62.45 to 63.39
Women		15 198	37.08	36.61 to 37.55
Age	40 986 (0.00)			
40–49		15 919	38.84	38.37 to 39.31
<29		5653	13.79	13.46 to 14.12
30–39		13 547	33.05	32.60 to 33.51
50–59		5207	12.70	12.39 to 13.03
>60		660	1.61	1.49 to 1.74
Marital status	40 828 (0.39)			
Married/cohabiting		31 710	77.67	77.26 to 78.07
Divorced/separated		3317	8.12	7.86 to 8.39
Single		4854	11.89	11.58 to 12.21
Other		947	2.32	2.18 to 2.47
Country	40 236 (1.83)			
England		28 465	70.75	70.30 to 71.19
Scotland		6428	15.98	15.62 to 16.34
Wales		5343	13.28	12.95 to 13.61
Education	40 828 (0.39)			
GSCE/O-level or below		13 675	33.49	33.04 to 33.95
A levels/higher or equivalent (NVQ3)		12 960	31.74	31.29 to 32.20
Bachelor degree/postgraduate qualifications		11 326	27.74	27.31 to 28.18
Vocational qualifications (NVQ1 + 2)		2867	7.02	6.78 to 7.27
Ethnicity	40 786 (0.49)			
White		38 643	94.75	94.52 to 94.96
Asian		690	1.69	1.57 to 1.82
Black		438	1.07	0.98 to 1.18
Mixed race		456	1.12	1.02 to 1.22
Other		559	1.37	1.26 to 1.49
Children under 18	40 828 (0.39)			
0		20 737	50.79	50.31 to 51.28
1		8139	19.93	19.55 to 20.33
2		9552	23.40	22.99 to 23.81
3 or more		2400	5.88	5.65 to 6.11
Years in police force	40 931 (0.13)			
11–20		12 679	30.98	30.53 to 31.43
Less than 5		8466	20.68	20.29 to 21.08
6–10		9231	22.55	22.15 to 22.96
More than 20		10 555	25.79	25.37 to 26.21
Role	37 127 (9.42)			
Police officer		26 031	70.11	69.65 to 70.58
Police staff		10 361	27.91	27.45 to 28.37
Other ranks		735	1.98	1.84 to 2.13
Income	40 828 (0.39)			
£26 000–37 999		16 820	41.20	40.72 to 41.68
Less than £25 999		8832	21.63	21.24 to 22.03
£38 000–59 999		13 869	33.97	33.51 to 34.43
More than £60 000		1307	3.20	3.03 to 3.38
Days of sickness	40 928 (0.14)			
None		18 956	46.32	45.83 to 46.80
1–5		13 504	32.99	32.54 to 33.45
6–10		3703	9.05	8.77 to 9.33
More than 10		4765	11.64	11.34 to 11.96
Smoking status	40 934 (0.13)			
Not smoker		36 902	90.15	89.86 to 90.43
Current smoker		4032	9.85	9.57 to 10.14

### Prevalence estimates

Prevalence estimates for the alcohol categories are outlined in Table 2. Most of the sample were low-risk drinkers (55.1%), with 32.6% meeting the criteria for hazardous use, and 3.0% for harmful use. When separated by gender, 19.4% of women and 40.4% of men were hazardous drinkers; 2.5% of women and 3.4% of men were harmful drinkers. A total of 9% reported abstinence (11.9% of women and 7.6% of men). For frequent binge drinking, 21.2% of women and 35.6% of men met criteria. Of the hazardous drinkers, 60.3% reported frequent binge drinking, with 88.9% of harmful drinkers frequently binge drinking (online Supplementary Table S1).

**Table 2.** Proportions and percentages for past week alcohol consumption, binge drinking, mental health and job strain

	Median units (IQR)	Total *N*	% (95% CI)	Men *N*	% (95% CI)	Women *N*	% (95% CI)
Summary of past week alcohol consumption (*N* *=* 40 986)							
Non-drinker	0	3764	9.18 (8.91 to 9.47)	1950	7.56 (7.25 to 7.89)	1814	11.94 (11.43 to 12.46)
Up to 14 units (low risk)	5.5 (2.5 to 9.5)	22 612	55.17 (54.69 to 55.65)	12 558	48.70 (48.09 to 49.31)	10 054	66.15 (65.40 to 66.90)
Between 14 and 35/50 units (hazardous)	22.5 (18.0 to 29.5)	13 365	32.61 (32.16 to 33.06)	10 412	40.38 (39.78 to 40.98)	2953	19.43 (18.80 to 20.07)
35+ units for women, 50+ units for men (harmful)	56.0 (50.5 to 66.0)	1245	3.04 (2.88 to 3.21)	868	3.37 (3.15 to 3.59)	377	2.48 (2.24 to 2.74)
Binge drinking (*N* *=* 40 986)							
Does not binge drink	5.5 (0.0 to 12.0)	28 577	69.72 (69.28 to 70.17)	16 598	64.36 (63.78 to 64.95)	11 979	78.82 (78.16 to 79.46)
Frequent binge drinking^a^	21.5 (14.0 to 32.0)	12 409	30.28 (29.83 to 30.72)	9190	35.64 (35.05 to 36.22)	3219	21.18 (20.54 to 21.84)
Mental health (*N* *=* 40 372)^b^							
Depression caseness (PHQ-9)	–	3958	9.80 (9.52 to 10.10)	2090	8.24 (7.91 to 8.58)	1868	12.45 (11.94 to 12.99)
Anxiety caseness (HADS)	–	3.407	8.44 (8.17 to 8.71)	1578	6.22 (5.93 to 6.52)	1829	12.19 (11.68 to 12.73)
PTSD caseness (BTS)	–	1520	3.95 (3.75 to 4.15)	979	4.05 (3.81 to 4.31)	541	3.76 (3.47 to 4.09)
Job strain (JCQ) (*N* *=* 40 372)							
Low	–	11 015	27.28 (26.85 to 27.72)	7515	29.62 (29.06 to 30.18)	3500	23.33 (22.66 to 24.02)
High	–	9722	24.08 (23.67 to 24.50)	5799	22.86 (22.34 to 23.38)	3923	26.16 (25.46 to 26.86)
Active	–	11 246	27.86 (27.42 to 28.30)	7454	29.38 (28.82 to 29.94)	3792	25.28 (24.59 to 25.98)
Passive	–	8389	20.78 (20.39 to 21.18)	4605	18.15 (17.68 to 18.62)	3784	25.23 (24.53 to 25.92)

IQR, interquartile range; PHQ-9, Patient Health Questionnaire-9 (Kroenke *et al.*, 2001); HADS, Hospital Anxiety and Depression Scale (anxiety sub-scale only) (Zigmond & Snaith, 1983); BTS, Brief Trauma Screen (Brewin *et al*., 2002); JCQ, 6 items from Job Content Questionnaire (Karasek, 1985).

a Frequent binge drinking defined as 6 or more units, at least 2 to 4 times a month.

b Total *N* for PTSD =  38 517.

The mental health and job strain characteristics of the sample are described in Table 2. In total, 9.8% met the criteria for probable depression, 8.4% for anxiety and 4% for PTSD. Around 27% were categorised as having low job strain, 24% as high job strain, 28% as active job strain and 21% were categorised as having passive job strain.

The prevalence estimates for the categories of alcohol consumption, probable mental health problems, job strain and key demographic variables (mean age, ethnicity and gender composition), separated by year of data collection (in 3–4 year bands), are presented and discussed in the online Supplementary materials. As the data were collected over a long period of time, these descriptive statistics can be used to cautiously observe cross-sectional trends. The limitations of this approach are outlined in the online Supplementary materials.

### Associations between mental health, job strain and alcohol use

Compared to those without a mental health problem, police employees with probable depression and anxiety were 1.3 times as likely to abstain from alcohol, with a weak association between PTSD and abstinence (Table 3). Those with depression, anxiety and PTSD were also twice as likely to drink harmfully and frequently binge drink (Table 4). All associations remained after adjusting for potential confounders, with the associations between anxiety and depression with harmful drinking/binge drinking, stronger after adjustments. Those with PTSD and anxiety were 1.3 times more likely to report hazardous drinking, with anxiety only becoming statistically significant after adjustments. After adjustment, police employees reporting low strain were significantly more likely to drink hazardously, or frequently binge drink, compared to those who reported high strain.

**Table 3.** Unadjusted and adjusted multinomial logistic regression analysis showing the associations between mental health and job strain, as explanatory variables and alcohol consumption as the outcome variable

	Non-drinkers			Low risk (ref)	Hazardous use			Harmful use		
Characteristic	*N* (%)	MOR (95% CI)	AMOR (95% CI)	*N* (%)	*N* (%)	MOR (95% CI)	AMOR (95% CI)	*N* (%)	MOR (95% CI)	AMOR (95% CI)
Mental health										
Depression case	474 (11.98)	1.41 (1.26 to 1.56)***	1.34 (1.20 to 1.49)***	2098 (53.01)	1160 (29.31)	0.93 (0.87 to 1.01)	1.04 (0.97 to 1.13)	226 (5.71)	2.18 (1.88 to 2.54)***	2.30 (1.97 to 2.69)***
Anxiety case	386 (11.33)	1.35 (1.20 to 1.52)***	1.32 (1.17 to 1.48)***	1757 (51.57)	1090 (31.99)	1.06 (0.98 to 1.15)	1.27 (1.17 to 1.38)***	174 (5.11)	1.94 (1.64 to 2.29)***	2.20 (1.85 to 2.61)***
PTSD case	155 (10.20)	1.29 (1.08 to 1.54)**	1.27 (1.06 to 1.52)*	725 (47.70)	556 (36.58)	1.34 (1.20 to 1.50)***	1.33 (1.18 to 1.49)***	84 (5.53)	2.28 (1.80 to 2.88)***	2.28 (1.80 to 2.89)***
Job strain										
Low	930 (8.44)	1.00	1.00	5896 (53.53)	3822 (34.70)	1.00	1.00	367 (3.33)	1.00	1.00
High	1013 (10.42)	1.15 (1.05 to 1.27)**	1.09 (0.98 to 1.20)	5.550 (57.09)	2885 (29.67)	0.80 (0.75 to 0.85)***	0.89 (0.84 to 0.95)***	274 (2.82)	0.79 (0.67 to 0.93)**	0.86 (0.73 to 1.01)
Active	992 (8.82)	1.04 (0.95 to 1.15)	1.01 (0.92 to 1.12)	6028 (53.60)	3886 (33.55)	0.99 (0.94 to 1.05)	0.99 (0.94 to 1.05)	340 (3.02)	0.91 (0.78 to 1.06)	0.89 (0.76 to 1.04)
Passive	785 (9.36)	1.03 (0.93 to 1.14)	0.96 (0.87 to 1.07)	4834 (57.62)	2528 (30.13)	0.81 (0.76 to 0.86)***	0.94 (0.88 to 1.00)	242 (2.88)	0.80 (0.68 to 0.95)**	0.90 (0.76 to 1.07)
Interactions^a^										
*Job strain × support*										
Low × support	–	1.00	–	–	–	1.00	–	–	1.00	–
High × support	–	0.97 (0.93 to 1.01)	–	–	–	1.01 (0.98 to 1.03)	–	–	1.00 (0.93 to 1.07)	–
Active × support	–	0.97 (0.93 to 1.01)	–	–	–	1.00 (0.97 to 1.03)	–	–	1.01 (0.94 to 1.08)	–
Passive × support	–	0.99 (0.95 to 1.03)	–	–	–	0.99 (0.96 to 1.02)	–	–	0.95 (0.89 to 1.02)	–
*Job strain* × *mental health*										
Low × MHC	96 (10.56)	1.00	–	479 (52.70)	290 (31.90)	1.00	–	44 (4.84)	1.00	–
High × MHC	291 (12.53)	1.09 (0.82 to 1.43)	–	1229 (52.93)	690 (29.70)	1.19 (0.99 to 1.43)	–	112 (4.82)	1.58 (1.04 to 2.38)*	–
Active × MHC	187 (10.57)	1.07 (0.80 to 1.43)	–	859 (48.56)	641 (36.24)	1.28 (1.06 to 1.55)*	–	82 (4.64)	1.24 (0.82 to 1.89)	–
Passive × MHC	140 (10.53)	0.95 (0.70 to 1.29)	–	724 (54.44)	396 (29.77)	1.14 (0.93 to 1.39)	–	70 (5.26)	1.50 (0.97 to 2.32)	–

MHC, mental health case.

Low risk drinking is the reference group. Row frequencies and percentages, with multinomial odds ratios (MOR) are shown.

*p < 0.05, **p < 0.01, ***p < 0.001.

Adjusted for age, gender, education, ethnicity, income, marital status, children under 18 and smoking status.

Reference groups for mental health are non-case.

a Support is a continuous variable; mental health is a categorical variable representing the presence of any mental health problem (depression, anxiety or PTSD) (case *v*. non-case).

**Table 4.** Unadjusted and adjusted multinomial logistic regression analysis showing the associations between mental health and job strain, as explanatory variables and binge drinking as the outcome variable

	Does not frequently binge drink	Frequently binge drinks		
Characteristic	*N* (%)	*N* (%)	OR (95% CI)	AOR (95% CI)
Mental health				
Depression case	2697 (9.57)	1261 (10.35)	1.09 (1.02 to 1.17)*	1.14 (1.06 to 1.23)***
Anxiety case	2320 (8.23)	1087 (8.92)	1.09 (1.01 to 1.18)*	1.23 (1.14 to 1.33)***
PTSD case	985 (3.65)	535 (4.64)	1.29 (1.15 to 1.43)***	1.27 (1.13 to 1.42)***
Job strain				
Low	7487 (26.56)	3528 (28.95)	1.00	1.00
High	6930 (24.59)	2792 (22.91)	0.85 (0.81 to 0.91)***	0.88 (0.83 to 0.94)***
Active	7734 (27.44)	3512 (28.82)	0.96 (0.91 to 1.02)	0.96 (0.91 to 1.02)
Passive	6033 (21.41)	2356 (19.33)	0.83 (0.78 to 0.88)***	0.90 (0.84 to 0.96)**
Interactions^a^				
*Job strain* × *support*				
Low × support	–	–	1.00	–
High × support	–	–	1.02 (0.99 to 1.05)	–
Active × support	–	–	1.02 (1.00 to 1.05)	–
Passive × support	–	–	0.99 (0.97 to 1.02)	–
*Job strain* × *mental health*				
Low × MHC	602 (66.23)	307 (30.58)	1.00	–
High × MHC	1612 (69.42)	710 (33.77)	1.03 (0.87 to 1.23)	–
Active × MHC	1162 (65.69)	607 (34.31)	1.08 (0.91 to 1.30)	–
Passive × MHC	931 (70.00)	399 (30.00)	1.02 (0.85 to 1.24)	–

MHC, mental health case.

Row frequencies and percentages are shown.

*p < 0.05, **p < 0.01, ***p < 0.001.

Adjusted for age, gender, education, ethnicity, income, marital status, children under 18 and smoking status.

Reference groups for mental health are non-case.

a Support is a continuous variable; mental health is a categorical variable representing the presence of any mental health problem (depression, anxiety or PTSD) (case *v*. non-case).

Exploratory sensitivity analysis, using only ‘non-drinkers’, identified that ‘former drinkers’ (responded ‘yes’ to ever drinking alcohol) were significantly more likely to report depression and anxiety than ‘never drinkers’ (responded ‘no’ to ever drinking alcohol), but the association with anxiety was not significant after adjustments (online Supplementary Table S2).

### Moderating effect of support or mental health on the association between job strain and alcohol use

The interaction term between support and job strain was not associated with the categories of alcohol use or binge drinking. The interaction term between mental health (meeting criteria for any mental health problem) and job strain was not associated with binge drinking but was significantly associated with the categories of alcohol use. Specifically, the interaction between mental health and active job strain was significantly associated with hazardous drinking, and the interaction between high job strain and mental health was significantly associated with harmful drinking.

To explore these interactions, the sample was stratified by the presence/absence of a mental health problem (Table 5). For those without a mental health problem, low strain (relative to high and passive strain) was associated with a greater risk of hazardous or harmful drinking, remaining significant after adjustments. For those with a mental health problem, active strain (relative to low strain) was associated with a greater risk of hazardous drinking, but this attenuated to the null after adjustment for confounders.

**Table 5.** Stratified by the presence of any mental health problem (mental health case *v*. mental health non-case), unadjusted and adjusted multinomial logistic regression analysis showing the associations between job strain and alcohol consumption

	Non-drinkers				Low risk		Hazardous use				Harmful use			
Characteristic	*N*	%	MOR (95% CI)	AMOR (95% CI)	*N*	%	*N*	%	MOR (95% CI)	AMOR (95% CI)	*N*	%	MOR (95% CI)	AMOR (95% CI)
*Mental health case*														
Job strain														
Low	96	10.56	1.00	1.00	479	52.70	290	31.90	1.00	1.00	44	4.84	1.00	1.00
High	291	12.53	1.18 (0.92 to 1.52)	1.18 (0.90 to 1.12)	1229	52.93	690	29.72	0.93 (0.78 to 1.10)	0.91 (0.76 to 1.09)	112	4.82	0.99 (0.69 to 1.43)	0.96 (0.66 to 1.39)
Active	187	10.57	1.08 (0.83 to 1.42)	1.08 (0.82 to 1.42)	859	48.56	641	36.24	1.23 (1.03 to 1.47)*	1.17 (0.97 to 1.41)	82	4.64	1.04 (0.71 to 1.52)	0.96 (0.65 to 1.41)
Passive	140	10.53	0.96 (0.73 to 1.28)	0.98 (0.73 to 1.30)	724	54.44	396	29.77	0.90 (0.75 to 1.09)	1.00 (0.82 to 1.22)	70	5.26	1.05 (0.71 to 1.56)	1.11 (0.75 to 1.67)
*Mental health non-case*														
Job strain														
Low	834	8.25	1.00	1.00	5417	53.60	3532	34.95	1.00	1.00	323	3.20	1.00	1.00
High	722	9.76	1.08 (0.97 to 1.21)	1.01 (0.90 to 1.12)	4321	58.39	2195	29.66	0.78 (0.73 to 0.83)***	0.87 (0.81 to 0.94)***	162	2.19	0.63 (0.52 to 0.76)***	0.70 (0.57 to 0.85)***
Active	805	8.49	1.01 (0.91 to 1.12)	0.98 (0.88 to 1.09)	5169	54.54	3245	34.24	0.96 (0.91 to 1.02)	0.96 (0.89 to 1.02)	258	2.72	0.84 (0.71 to 0.99)*	0.83 (0.70 to 0.98)*
Passive	645	9.14	1.02 (0.91 to 1.14)	0.95 (0.85 to 1.06)	4110	58.22	2132	30.20	0.80 (0.74 to 0.85)***	0.92 (0.86 to 0.99)*	172	2.44	0.70 (0.58 to 0.85)***	0.80 (0.66 to 0.96)*

Low risk drinking is the reference group. Row frequencies and percentages, with multinomial odds ratios (MOR) are shown.

*p < 0.05, ***p < 0.001.

Adjusted for age, gender, education, ethnicity, income, marital status, children under 18 and smoking status.

### Exploratory associations between sociodemographic, occupational and health variables with alcohol use

Men were more likely than women to report hazardous and harmful drinking (online Supplementary Table S3). Those aged 40–49 (reference) were more likely to drink hazardously than any other age group, and more likely to drink harmfully than those under 39. Being single (reference: married) was associated with harmful drinking. Those who had served for 11–20 years (reference) were more likely to be hazardous or harmful drinkers than those who had served for less than 10 years, but less likely than those who had served for over 20 years. Police officers (reference) were more likely to be hazardous or harmful drinkers than police staff. Those with more than 10 days of sickness (reference: none) were more likely to drink harmfully but also more likely to report abstinence. Being a current smoker was associated with almost three times greater odds of harmful alcohol use. Similar associations were observed with binge drinking as the outcome (online Supplementary Table S4).

## Discussion

### Key findings

This is the first and largest study of alcohol use in the UK Police Service. Approximately one-third of police employees reported hazardous drinking and frequent binge drinking, with 3% drinking at harmful levels. Men were more likely than women to report hazardous and harmful drinking, reflecting findings from the general population (NHS Digital, 2018). Police employees with a probable mental health problem were more likely to report harmful drinking, but were also more likely to report abstinence. Opposing our hypothesis, those reporting low strain drank more than those reporting high strain, but when stratified by mental health, this association was only shown in police employees without a mental health problem.

The prevalence of hazardous drinking was higher in the UK Police Service than the general population, for men (40 *v*. 24%) and women (19 *v*. 11%) (NHS Digital, 2018), although the level of harmful drinking is similar. However, this is not a direct comparison with the UK general population data and there may be considerable differences between samples (e.g. age, year of data collection). The present findings reflect those observed in a representative sample of Australian police officers (Davey, 2000), whereby men reported more hazardous drinking than women, but showed comparable levels of harmful drinking (approximately 3%). However, the US literature shows much lower levels of hazardous drinking in police officers and little difference between genders, e.g. 17% (Lindsay, 2008), 18% (Chopko *et al*., 2013), 14% of men and 12% of women (Ménard and Arter, 2014), 18% of men and 16% of women (Ballenger *et al*., 2011). Nevertheless, there are cultural differences in alcohol consumption between the UK and USA (Ritchie and Roser, 2018).

### Alcohol use and the associations with mental health and job strain

Police employees with probable depression, anxiety or PTSD were twice as likely to report harmful alcohol use and frequent binge drinking, compared to those without a mental health problem, but were also more likely to abstain from alcohol. This is in line with findings from a global study whereby low-risk drinking, compared to abstinence, was associated with lower depression and anxiety, but harmful drinking was associated with greater levels of depression and anxiety (Bellos *et al*., 2013). Similar findings have been observed in military personnel, but for PTSD only (Goodwin *et al*., 2017). Taken together, these findings suggest a J-shaped curve in the relationship between mental health and alcohol use, whereby positive self-reports of mental health are associated with low-risk drinking, but not heavy drinking or abstinence (El-Guebaly, 2007). There are mental health differences between ‘lifestyle choice abstainers’ and ‘previous problem drinking abstainers’ (El-Guebaly, 2007), with our exploratory analyses suggesting that former drinkers were more likely to report depression compared to those who have never drank. It may be that police employees who now abstain from alcohol do so because of a previous alcohol problem or because alcohol was poorly affecting their mental health. However, longitudinal, and qualitative data are needed to better understand the causal relationship.

Few studies have explored the relationship between mental health and alcohol use in police officers, with the few focussing on PTSD. The prevalence of PTSD is lower in the current study than other UK and international studies of police officers (Skogstad *et al*., 2013; Violanti *et al*., 2017). However, the TSQ was only administered if participants reported experiencing a traumatic event in the past 6 months, excluding those with delayed onset PTSD (>6 months) or earlier trauma exposure (Utzon-Frank *et al*., 2014). An abundance of literature has shown a relationship between mental health and alcohol problems (McFarlane, 1998; Debell *et al*., 2014), with the self-medication hypothesis suggesting that alcohol is used as a form of avoidance coping to alleviate negative affect (Khantzian, 1997; Stewart *et al*., 2004). The existing evidence on PTSD and alcohol use in police officers is mixed, with some studies showing an association (Chopko *et al*., 2013; Ménard and Arter, 2014), whereas others do not (Ballenger *et al*., 2011; Violanti *et al*., 2011). One study noted that the combined effect of heavy drinking and PTSD led to a ten-fold greater risk of suicide ideation in police officers (Violanti, 2004). A qualitative exploration of mental health in five police officers observed that some used alcohol to cope with work pressures and psychological symptoms, and reported that ‘macho’ police culture and stigma are barriers to help-seeking (Edwards and Kotera, 2020).

Opposing our hypothesis, police employees reporting low strain were more likely to drink hazardously than those reporting high strain, remaining significant after adjustment for indicators of seniority (e.g. income and years of service). However, when stratified by mental health, the association was only apparent in those without a mental health problem. Previous research in police officers is mixed, with some studies showing an association between work stress and hazardous drinking (Violanti *et al*., 1985; Kohan and O'connor, 2002; Violanti *et al*., 2011), whereas others do not (Sterud *et al*., 2007). Furthermore, there is inconsistent evidence that alcohol is used to self-medicate negative affect caused by work stressors (Frone, 1999; Siegrist and Rödel, 2006). The biphasic self-medication model suggests that work stressors increase negative affect, which initially leads to higher alcohol use in those with higher stress, but the sedative effects of alcohol increase negative affect and work fatigue, making this pattern of behaviour difficult to maintain (Frone, 2016). Those with low strain may experience lower negative affect (or better mental health) from work stress, and have more time to socialise, and therefore drink more, due to holding less senior job roles.

### Strengths and limitations

The main strength of this study is that it utilises a large sample of police employees. Both men and women were recruited, across all regions of the UK, including large numbers of men aged between 20 and 40, who are often under-represented in other epidemiological studies (Medical Research Council, 2014). The study was originally designed to measure the physical effects of TETRA radio usage, rather than alcohol use or mental health, reducing bias from framing effects (Tversky and Kahneman, 1981; Goodwin *et al*., 2013). Nevertheless, there are caveats. Alcohol consumption was measured using a 7-day drinks diary, which may be subject to recall bias, and the TSQ was only asked to those who reported experiencing a traumatic event in the past 6 months. A further limitation is that we were unable to distinguish across different police roles, such as constables and sergeants, as participants were grouped into ‘police officers’, ‘police staff’ and ‘other’ ranks of police employees, for identification purposes. Finally, as this study is cross-sectional, we were unable to determine the causal relationship between alcohol consumption and mental health.

### Implications

Identifying occupational groups at a higher risk of alcohol harm enables us to develop targeted interventions within that occupation, such as those being trialled in military personnel (Leightley *et al*., 2018). Evidence from military personnel and first responders shows low levels of help-seeking for alcohol problems, due to stigma (Sharp *et al*., 2015; Haugen *et al*., 2017). Education on low-risk drinking should be implicated within police workforces and avenues for help-seeking should be made easily available, without disciplinary action (Home Office, 2012). The present findings show that police employees with a mental health problem were more likely to drink harmfully. Longitudinal and qualitative research is needed to explore whether alcohol is used as a coping mechanism within police employees, and to examine trends in the relationship between alcohol use and strain over time, particularly following changes to workforce pressures (e.g. budget cuts).

## Conclusions

One-third of UK police employees drink hazardously and binge drink at least twice a month. Furthermore, there is evidence for a J-shaped relationship between alcohol use and mental health in UK police employees, with those reporting mental health problems being more likely to report both abstinence and harmful drinking. Interventions to reduce risky drinking within police employees should also integrate support for mental health.

## Supplementary material

For supplementary material accompanying this paper visit http://dx.doi.org/10.1017/S2045796021000366. click here to view supplementary material

## Availability of data and materials

Due to confidentiality, we are not able to share the data. Access to the Airwave Health Monitoring Study Data must be obtained through a formal application process (https://police-health.org.uk/applying-access-resource).
