# Supplementary material for: The prevalence of hazardous and harmful drinking in the UK Police Service, and their co-occurrence with job strain and mental health problems
Source: Epidemiol Psychiatr Sci. 2021 Jun 21;30:e51. doi: 10.1017/S2045796021000366 (PMC8220482; doi:10.1017/S2045796021000366)
Supplement: Supplementary file 1 [file S2045796021000366sup001.docx]

**Table S1.** Frequencies and percentages for low risk, hazardous and harmful drinking, separated by binge drinking ^a^.

|  | **Total = 40,986** | | **Does not frequently binge drin**k **(N = 28,577)** | | **Frequent binge drinking**  **(N = 12,390)** | |
| --- | --- | --- | --- | --- | --- | --- |
|  | **N** | **% (95% CI)** | **N** | **% (95% CI)** | **N** | **% (95% CI)** |
| Non-drinker | 3,764 | 9.18 (8.91 to 9.47) | 3,764 | 100.00 | - | - |
| Up to 14 units (low risk) | 22,612 | 55.17 (54.69 to 55.65) | 19,362 | 85.63 (85.16 to 86.08) | 3,250 | 14.37 (13.92 to 14.83) |
| Between 14 and 35/50 units (hazardous) | 13,365 | 32.61 (32.16 to 33.06) | 5,313 | 39.75 (38.93 to 40.59) | 8,052 | 60.25 (59.41 to 61.07) |
| 35+ units for women, 50+ units for men (harmful) | 1,245 | 3.04 (2.87 to 3.20) | 138 | 11.08 (9.46 to 12.95) | 1,107 | 88.92 (87.05 to 90.54) |

^a^ frequent binge drinking defined as 6 or more units, at least 2 to 4 times a month

**Table S2.** Exploratory logistic regression analyses examining differences in depression, anxiety and PTSD in abstainers who were former drinkers, versus abstainers who have never drank.

| **Characteristic** | | **Never drinker** | **Former drinker** | | |
| --- | --- | --- | --- | --- | --- |
|  | | **N (%)** | **N (%)** | **OR (95% CI)** | **AOR (95% CI)** |
|  | Depression non-case | 988 (89.49) | 2,258 (86.31) | 1.00 | 1.00 |
|  | Depression case | 116 (10.51) | 358 (13.69) | 1.35 (1.08 to 1.69)* | 1.34 (1.06 to 1.70)* |
|  | Anxiety non-case | 1,009 (91.39) | 2,325 (88.88) | 1.00 | 1.00 |
|  | Anxiety case | 95 (8.61) | 291 (11.12) | 1.33 (1.04 to 1.70)* | 1.21 (0.94 to 1.57) |
|  | PTSD non-case | 1,019 (96.40) | 2,404 (95.36) | 1.00 | 1.00 |
|  | PTSD case | 38 (3.60) | 117 (4.64) | 1.30 (0.90 to 1.90) | 1.25 (0.84 to 1.86) |

*p < .05, **p < .01, ***p < .001

Adjusted for age, gender, education, ethnicity, income, marital status, children under 18, and smoking status.

**Table S3.** Multinomial logistic regression analysis, adjusted for age and gender, showing the associations between sociodemographic, occupational and health factors with alcohol consumption as the outcome. Low-risk drinking is the reference group. Row frequencies and percentages, with adjusted multinomial odds ratios (AMOR) are shown.

| **Characteristic** | | **Non-drinker** | | | **Low risk** | | **Hazardous use** | | | **Harmful use** | | |
| --- | --- | --- | --- | --- | --- | --- | --- | --- | --- | --- | --- | --- |
|  | | **N (%)** | | **AMOR (95% CI)** | **N (%)** | | **N (%)** | | **AMOR (95% CI)** | **N (%)** | | **AMOR (95% CI)** |
| Gender | |  |  |  |  |  |  |  |  |  |  |  |
|  | Women | 1,814 (11.94) | | 1.00 | 10,054 (66.15) | | 2,953 (19.43) | | 1.00 | 377 (2.48) | | 1.00 |
|  | Men | 1,950 (7.56) | | 0.85 (0.80 to 0.92)*** | 12,558 (48.70) | | 10,412 (40.38) | | 2.74 (2.61 to 2.87)*** | 868 (3.37) | | 1.75 (1.54 to 1.98)*** |
| Age | |  |  |  |  |  |  |  |  |  |  |  |
|  | 40 to 49 | 1,311 (8.24) | | 1.00 | 8,205 (51.54) | | 5,824 (36.59) | | 1.00 | 579 (3.64) | | 1.00 |
|  | <29 | 531 (9.39) | | 0.91 (0.81 to 1.01) | 3,572 (63.19) | | 1,440 (25.47) | | 0.66 (0.61 to 0.71)*** | 110 (1.95) | | 0.48 (0.39 to 0.59)*** |
|  | 30 to 39 | 1,348 (9.95) | | 1.09 (1.00 to 1.18)* | 7,679 (53.35) | | 4,162 (30.72) | | 0.81 (0.77 to 0.86)*** | 358 (2.64) | | 0.69 (0.60 to 0.79)*** |
|  | 50 to 59 | 496 (9.53) | | 1.12 (1.00 to 1.25) | 2,778 (53.35) | | 1,752 (33.65) | | 0.90 (0.84 to 0.96)** | 181 (3.48) | | 0.93 (0.78 to 1.10) |
|  | >60 | 78 (11.82) | | 1.29 (0.98 to 1.66) | 378 (57.27) | | 187 (28.33) | | 0.70 (0.59 to 0.84)*** | 17 (2.58) | | 0.64 (0.39 to 1.05) |
| Marital status | |  |  |  |  |  |  |  |  |  |  |  |
|  | Married/Cohabiting | 2,844 (8.97) | | 1.00 | 17,268 (54.46) | | 10,645 (33.57) | | 1.00 | 953 (3.01) | | 1.00 |
|  | Divorced/Separated | 292 (8.80) | | 0.93 (0.82 to 1.06) | 1,835 (55.32) | | 1,074 (32.38) | | 1.06 (0.98 to 1.15) | 116 (3.50) | | 1.17 (0.95 to 1.42) |
|  | Single | 530 (10.92) | | 1.13 (1.02 to 1.26)* | 2,868 (59.09) | | 1,314 (27.07) | | 1.06 (0.99 to 1.14) | 142 (2.93) | | 1.27 (1.05 to 1.53)* |
|  | Other | 87 (9.19) | | 0.96 (0.76 to 1.20) | 552 (58.29) | | 279 (29.46) | | 1.12 (0.96 to 1.30) | 29 (3.06) | | 1.28 (0.87 to 1.88) |
| Country | |  |  |  |  |  |  |  |  |  |  |  |
|  | England | 2,806 (9.86) | | 1.00 | 15,782 (55.44) | | 9,031 (31.73) | | 1.00 | 846 (2.97) | | 1.00 |
|  | Scotland | 461 (7.17) | | 0.73 (0.66 to 0.81)*** | 3,595 (55.93) | | 2,198 (34.19) | | 1.02 (0.96 to 1.09) | 174 (2.71) | | 0.88 (0.75 to 1.04) |
|  | Wales | 431 (8.07) | | 0.86 (0.77 to 0.96)** | 2,816 (52.70) | | 1,895 (35.47) | | 1.25 (1.17 to 1.34)*** | 201 (3.76) | | 1.41 (1.21 to 1.66)*** |
| Education | |  |  |  |  |  |  |  |  |  |  |  |
|  | GSCE/O-Level or below | 1,298 (9.49) | | 1.00 | 7,297 (53.36) | | 4,595 (33.60) | | 1.00 | 485 (3.55) | | 1.00 |
|  | Vocational qualifications | 285 (9.94) | | 0.99 (0.86 to 1.14) | 1,622 (56.57) | | 896 (31.25) | | 0.94 (0.86 to 1.03) | 64 (2.23) | | 0.65 (0.50 to 0.86)** |
|  | A levels or equivalent | 1,149 (8.87) | | 0.91 (0.83 to 0.99)* | 7,199 (55.55) | | 4,199 (32.40) | | 1.00 (0.95 to 1.05) | 413 (3.19) | | 0.96 (0.84 to 1.10) |
|  | Bachelor Degree/Postgraduate | 1,021 (9.01) | | 0.90 (0.82 to 0.99)* | 6,405 (56.55) | | 3,622 (31.98) | | 1.00 (0.94 to 1.06) | 278 (2.45) | | 0.76 (0.65 to 0.89)*** |
| Ethnicity | |  |  |  |  |  |  |  |  |  |  |  |
|  | White | 3,151 (8.15) | | 1.00 | 21,396 (55.37) | | 12,889 (33.35) | | 1.00 | 1,207 (3.12) | | 1.00 |
|  | Asian | 317 (45.94) | | 7.35 (6.25 to 8.66)*** | 297 (43.04) | | 69 (10.00) | | 0.37 (0.28 to 0.48)*** | 7 (1.01) | | 0.42 (0.20 to 0.90)* |
|  | Black | 134 (30.59) | | 3.39 (2.74 to 4.19)*** | 261 (59.59) | | 39 (8.90) | | 0.26 (0.18 to 0.36)*** | 4 (0.91) | | 0.26 (0.10 to 0.71)** |
|  | Mixed Race | 53 (11.62) | | 1.35 (1.01 to 1.82)* | 271 (59.43) | | 122 (26.75) | | 0.74 (0.59 to 0.92)** | 11 (2.19) | | 0.67 (0.35 to 1.26) |
|  | Other | 88 (15.74) | | 2.21 (1.73 to 2.82)*** | 279 (49.91) | | 180 (32.20) | | 0.91 (0.75 to 1.10) | 12 (2.15) | | 0.68 (0.38 to 1.21) |
| Children Under 18 | |  |  |  |  |  |  |  |  |  |  |  |
|  | 0 | 1,906 (9.19) | | 1.00 | 11,541 (55.65) | | 6,623 (31.94) | | 1.00 | 667 (3.22) | | 1.00 |
|  | 1 | 797 (9.79) | | 1.08 (0.98 to 1.18) | 4,567 (56.11) | | 2,560 (31.45) | | 0.82 (0.77 to 0.87)*** | 215 (2.64) | | 0.69 (0.58 to 0.81)*** |
|  | 2 | 813 (8.51) | | 0.98 (0.89 to 1.07) | 5,170 (54.12) | | 3,279 (34.33) | | 0.85 (0.81 to 0.90)*** | 290 (3.04) | | 0.76 (0.66 to 0.89)*** |
|  | 3 or more | 237 (9.88) | | 1.20 (1.03 to 1.40)* | 1,245 (51.88) | | 850 (35.42) | | 0.86 (0.78 to 0.95)** | 68 (2.83) | | 0.70 (0.54 to 0.91)** |
| Years in police force | |  |  |  |  |  |  |  |  |  |  |  |
|  | 11 to 20 | 1,130 (8.91) | | 1.00 | 6,783 (53.50) | | 4,343 (34.25) | | 1.00 | 423 (3.34) | | 1.00 |
|  | Less than 5 | 839 (9.91) | | 1.02 (0.92 to 1.14) | 5,398 (62.17) | | 2,207 (26.07) | | 0.81 (0.76 to 0.88)*** | 157 (1.85) | | 0.57 (0.46 to 0.70)*** |
|  | 6 to 10 | 1,047 (11.34) | | 1.20 (1.09 to 1.32)*** | 5,263 (58.48) | | 2,555 (27.68) | | 0.81 (0.76 to 0.86)*** | 231 (2.50) | | 0.75 (0.63 to 0.89)** |
|  | More than 20 | 740 (7.01) | | 0.84 (0.76 to 0.94)** | 5,144 (48.74) | | 4,238 (40.15) | | 1.17 (1.10 to 1.25)*** | 433 (4.10) | | 1.22 (1.04 to 1.42)* |
| Role | |  |  |  |  |  |  |  |  |  |  |  |
|  | Police Officer | 2,050 (7.88) | | 1.00 | 13,645 (52.42) | | 9,476 (36.40) | | 1.00 | 954 (3.66) | | 1.00 |
|  | Police Staff | 1,270 (12.26) | | 1.31 (1.21 to 1.43)*** | 6,333 (61.12) | | 2,504 (28.16) | | 0.79 (0.74 to 0.84)*** | 266 (2.57) | | 0.77 (0.65 to 0.90)*** |
|  | Other | 91 (12.38) | | 1.44 (1.14 to 1.83)** | 411 (55.92) | | 207 (24.17) | | 0.88 (0.74 to 1.05) | 28 (3.81) | | 1.10 (0.73 to 1.66) |
| Income | |  |  |  |  |  |  |  |  |  |  |  |
|  | £26000 - £37999 | 1,593 (9.47) | | 1.00 | 9,438 (56.11) | | 5,306 (31.55) | | 1.00 | 483 (2.87) | | 1.00 |
|  | Less than £25999 | 1,065 (12.06) | | 1.08 (0.99 to 1.19) | 5,703 (64.57) | | 1,898 (21.49) | | 0.83 (0.78 to 0.89)*** | 166 (1.88) | | 0.65 (0.54 to 0.79)*** |
|  | £38000 - £59999 | 1,018 (7.34) | | 0.89 (0.81 to 0.97)* | 6,775 (48.85) | | 5,527 (39.85) | | 1.18 (1.12 to 1.24)*** | 549 (3.96) | | 1.32 (1.16 to 1.51)*** |
|  | More than £60000 | 77 (5.89) | | 0.75 (0.58 to 0.96)* | 607 (46.44) | | 607 (44.45) | | 1.32 (1.17 to 1.49)*** | 42 (3.21) | | 1.05 (0.75 to 1.46) |
| Days of sickness | |  |  |  |  |  |  |  |  |  |  |  |
|  | None | 1,614 (8.510 | | 1.00 | 10,255 (54.10) | | 6,532 (34.46) | | 1.00 | 555 (2.93) | | 1.00 |
|  | 1 to 5 | 1,176 (8.71) | | 0.95 (0.88 to 1.03) | 7,742 (57.33) | | 4,192 (31.04) | | 0.97 (0.93 to 1.02) | 394 (2.92) | | 1.06 (0.93 to 1.22) |
|  | 6 to 10 | 376 (10.15) | | 1.13 (1.00 to 1.28) | 2,073 (55.98) | | 1,147 (30.97) | | 1.01 (0.94 to 1.10) | 107 (2.89) | | 1.08 (0.87 to 1.34) |
|  | More than 10 | 596 (12.51) | | 1.47 (1.32 to 1.63)*** | 2,514 (52.76) | | 1,468 (30.81) | | 1.07 (1.00 to 1.15) | 187 (3.92) | | 1.52 (1.28 to 1.81)*** |
| Smoking status | |  |  |  |  |  |  |  |  |  |  |  |
|  | Not smoker | 3,384 (9.17) | | 1.00 | 20,704 (56.11) | | 11,798 (31.97) | | 1.00 | 1,016 (2.75) | | 1.00 |
|  | Current smoker | 375 (9.30) | | 1.21 (1.08 to 1.36)** | 1,876 (46.53) | | 1,553 (38.52) | | 1.62 (1.51 to 1.74)*** | 228 (5.65) | | 2.71 (2.33 to 3.16)*** |

*p < .05, **p < .01, ***p < .001

**Table S4.** Multinomial logistic regression analysis, adjusted for age and gender, showing the associations between sociodemographic, occupational and health factors with frequent binge drinking as the outcome. Row frequencies and percentages, with adjusted odds ratios (AOR) are shown.

| **Characteristic** | | **Does not frequently binge drink** | | **Frequent binge drinking** | | |
| --- | --- | --- | --- | --- | --- | --- |
|  | | **N (%)** | | **N (%)** | | **AOR (95% CI)** |
| Gender | |  |  |  |  |  |
|  | Women | 11,979 (78.82) | | 3,219 (21.18) | | 1.00 |
|  | Men | 16,598 (64.36) | | 9,190 (35.64) | | 2.08 (1.98 to 2.18)*** |
| Age | |  |  |  |  |  |
|  | 40 to 49 | 10,779 (67.71) | | 5,140 (32.29) | | 1.00 |
|  | <29 | 3,911 (69.18) | | 1,742 (30.82) | | 1.06 (0.99 to 1.13) |
|  | 30 to 39 | 9,598 (70.85) | | 3,949 (29.15) | | 0.91 (0.86 to 0.96)*** |
|  | 50 to 59 | 3,762 (72.25) | | 1,445 (27.75) | | 0.81 (0.76 to 0.87)*** |
|  | >60 | 527 (79.85) | | 133 (20.15) | | 0.53 (0.44 to 0.65)*** |
| Marital status | |  |  |  |  |  |
|  | Married/Cohabiting | 22,333 (70.43) | | 9,377 (29.57) | | 1.00 |
|  | Divorced/Separated | 2,271 (68.47) | | 1,046 (31.53) | | 1.24 (1.14 to 1.34)*** |
|  | Single | 3,211 (66.15) | | 1,643 (33.85) | | 1.49 (1.39 to 1.59)*** |
|  | Other | 651 (68.74) | | 296 (31.26) | | 1.29 (1.12 to 1.49)** |
| Country | |  |  |  |  |  |
|  | England | 20,410 (71.70) | | 8,055 (28.30) | | 1.00 |
|  | Scotland | 4,143 (64.45) | | 2,285 (35.55) | | 1.35 (1.27 to 1.43)*** |
|  | Wales | 3,502 (65.54) | | 1,841 (34.46) | | 1.36 (1.28 to 1.45)*** |
| Education | |  |  |  |  |  |
|  | GSCE/O-Level or below | 9,378 (68.58) | | 4,297 (31.42) | | 1.00 |
|  | Vocational qualifications | 2,018 (70.39) | | 849 (29.61) | | 0.92 (0.84 to 1.01) |
|  | A levels or equivalent | 9,004 (69.48) | | 3,956 (30.52) | | 0.95 (0.90 to 1.00)* |
|  | Bachelor Degree/Postgraduate | 8,066 (71.22) | | 3,260 (28.78) | | 0.88 (0.83 to 0.93)*** |
| Ethnicity | |  |  |  |  |  |
|  | White | 26,663 (69.00) | | 11,980 (31.00) | | 1.00 |
|  | Asian | 620 (89.86) | | 70 (10.14) | | 0.24 (0.19 to 0.31)*** |
|  | Black | 402 (91.78) | | 36 (8.22) | | 0.22 (0.15 to 0.31)*** |
|  | Mixed Race | 339 (74.34) | | 117 (25.66) | | 0.74 (0.60 to 0.92)** |
|  | Other | 404 (72.27) | | 155 (27.73) | | 0.76 (0.63 to 0.92)** |
| Children Under 18 | |  |  |  |  |  |
|  | 0 | 14,169 (68.33) | | 6,568 (31.67) | | 1.00 |
|  | 1 | 5,900 (72.49) | | 2,239 (27.51) | | 0.72 (0.67 to 0.76)*** |
|  | 2 | 6,731 (70.47) | | 2,821 (29.53) | | 0.75 (0.71 to 0.79)*** |
|  | 3 or more | 1,666 (69.42) | | 734 (30.58) | | 0.75 (0.68 to 0.83)*** |
| Years in police force | |  |  |  |  |  |
|  | 11 to 20 | 8,759 (69.08) | | 3,920 (30.92) | | 1.00 |
|  | Less than 5 | 6,072 (71.72) | | 2,394 (28.28) | | 0.86 (0.82 to 0.95)** |
|  | 6 to 10 | 6,789 (73.55) | | 2,442 (26.45) | | 0.80 (0.75 to 0.85)*** |
|  | More than 20 | 6,921 (65.57) | | 3,634 (34.43) | | 1.18 (1.11 to 1.25)*** |
| Role | |  |  |  |  |  |
|  | Police Officer | 17,435 (66.98) | | 8,596 (24.74) | | 1.00 |
|  | Police Staff | 7,798 (75.26) | | 2,563 (33.02) | | 0.96 (0.81 to 1.14) |
|  | Other | 536 (72.93) | | 199 (27.07) | | 0.89 (0.84 to 0.94)*** |
| Income | |  |  |  |  |  |
|  | £26000 - £37999 | 11,740 (69.80) | | 5,080 (30.20) | | 1.00 |
|  | Less than £25999 | 6,791 (76.89) | | 2,041 (23.11) | | 0.88 (0.83 to 0.94)*** |
|  | £38000 - £59999 | 9,088 (65.53) | | 4,781 (34.47) | | 1.11 (1.05 to 1.17)*** |
|  | More than £60000 | 847 (64.80) | | 460 (35.20) | | 1.14 (1.01 to 1.28)* |
| Days of sickness | |  |  |  |  |  |
|  | None | 13,095 (69.08) | | 5,861 (30.92) | | 1.00 |
|  | 1 to 5 | 9,502 (70.36) | | 4,002 (29.64) | | 1.01 (0.97 to 1.07) |
|  | 6 to 10 | 2,583 (69.75) | | 1,120 (30.25) | | 1.07 (0.99 to 1.18) |
|  | More than 10 | 3,360 (70.51) | | 1,405 (29.49) | | 1.05 (0.98 to 1.13) |
| Smoking status | |  |  |  |  |  |
|  | Not smoker | 26,185 (70.96) | | 10,717 (29.04) | | 1.00 |
|  | Current smoker | 2,349 (58.26) | | 1,683 (41.74) | | 1.87 (1.75 to 2.00)*** |

*p < .05, **p < .01, ***p < .001

**Exploratory cross-sectional analysis of trends in prevalence estimates over the period of data collection.**

Participants from the Airwave Health Monitoring Study were recruited over a nine year period, meaning there may be changes in prevalence estimates over time, due to changes such as budget cuts which reduced officer numbers from 2010, or in line with the downward trend of alcohol consumption observed in the general population (Office for National Statistics, 2018). Supplementary table 5 shows exploratory descriptive statistics for key demographic variables of interest, as well as the main outcome and explanatory variables (categories of alcohol consumption, probable mental health problems, and job strain), separated by year of data collection (in 3-4 year bands).

Supplementary table 5 shows a decrease in prevalence estimates for hazardous and harmful drinking, and an increase in the proportion of abstainers. The changes in alcohol consumption could relate to the changing demographics of the sample, as the proportion of participants from ethnic minority backgrounds, and the proportion of women, increased substantially during 2013-2015. Supplementary table 3 shows that participants from ethnic minority backgrounds had significantly higher odds of abstaining, compared to those of White ethnicity. Women were also significantly more likely to report abstinence than men. This could also reflect the trends observed in the general population, as there has been an increase in the proportion of adults reporting abstinence, in the past decade (Office for National Statistics, 2018). Further, the reduction in the proportion of hazardous and harmful drinkers could relate to changes to the drinking culture within policing, but further qualitative research is needed to explore this.

Supplementary table 5 also shows a slight increase in the proportion of police employees meeting criteria for high job strain (high demands, low control) and active strain (high demands, high control), but a decrease in participants reporting low strain (low demands, high control) in 2013-2015, which may reflect increasing levels of demands following the budget cuts which occurred in 2010.

There are considerable sampling and demographic differences which could influence prevalence estimates, meaning the cross-sectional trends should be interpreted cautiously. The original study protocol includes a supplementary table providing substantial detail regarding the recruitment procedure, showing that entire regions were recruited during certain timepoints. For example, all participants from the Metropolitan police service were recruited after 2011 (Elliott et al., 2014). There may be regional differences in the outcomes of interest (Robinson et al., 2015), therefore, the prevalence estimates separated by year of data collection may not be reliable. Nevertheless, the prevalence estimates for the full sample, for the categories of alcohol consumption and probable mental health problems, do not largely differ from the prevalence estimates across each of the year groups.

**Table S5.** Exploratory descriptive statistics for key demographic variables, alcohol, mental health, and job strain, separated by year of data collection, to explore trends over time.

|  |  | **2006-2009**  **N = 16,995 (41.47%)** | **2010-2012**  **N = 13,748 (33.54%)** | **2013-2015**  **N = 10,243 (24.99%)** | **Total**  **N = 40,986** |
| --- | --- | --- | --- | --- | --- |
| Mean age (±SD) | | 39.98 (±9.13) | 40.51 (±8.92) | 41.52 (±8.49) | 40.55 (±8.93) |
| Proportion White ethnicity | | 96.46% | 96.45% | 87.77% | 94.28% |
| Proportion of men | | 62.02% | 61.69% | 65.68% | 62.92% |
| Alcohol consumption | |  |  |  |  |
|  | Non-drinkers | 1,343 (7.90%) | 1,213 (8.82%) | 1,208 (11.79%) | 3,764 (9.18%) |
|  | Low-risk | 9,132 (53.73%) | 7,822 (56.90%) | 5,658 (55.24%) | 22,612 (55.17%) |
|  | Hazardous | 5,908 (34.76%) | 4,350 (31.64%) | 3,107 (30.33%) | 13,365 (32.61%) |
|  | Harmful | 612 (3.60%) | 363 (2.64%) | 270 (2.64%) | 1,245 (3.04%) |
| Mental health case-ness | |  |  |  |  |
|  | Depression case | 1,666 (10.14%) | 1,299 (9.46%) | 993 (9.72%) | 3,958 (9.80%) |
|  | Anxiety case | 1,494 (9.09%) | 1,116 (8.13%) | 797 (7.80%) | 3,407 (8.44%) |
|  | PTSD case ^a^ | 606 (4.15%) | 513 (3.87%) | 383 (3.75%) | 1,520 (3.95%) |
| Job strain | |  |  |  |  |
|  | Low | 4,590 (27.94%) | 4,064 (29.61%) | 2,361 (23.11%) | 11,015 (27.28%) |
|  | High | 3,830 (23.32%) | 3,052 (22.23%) | 2,840 (27.79%) | 9,722 (24.08%) |
|  | Passive | 5,017 (30.54%) | 3,612 (26.31%) | 2,617 (25.61%) | 11,246 (27.86%) |
|  | Active | 2,990 (18.20%) | 2,999 (21.85%) | 2,400 (23.49%) | 8,389 (20.78%) |

^a^ PTSD items not asked in 2006 version of protocol.
